# Supplementary material for: Equine Colostrum-Derived Mesenchymal Stromal Cells: A Potential Resource for Veterinary Regenerative Medicine
Source: Vet Sci. 2025 Jul 19;12(7):681. doi: 10.3390/vetsci12070681 (PMC12300667; doi:10.3390/vetsci12070681)
Supplement: Supplementary file 1 [file vetsci-12-00681-s001.zip › vetsci-3590252-supplementary.pdf]

Supplementary Materials

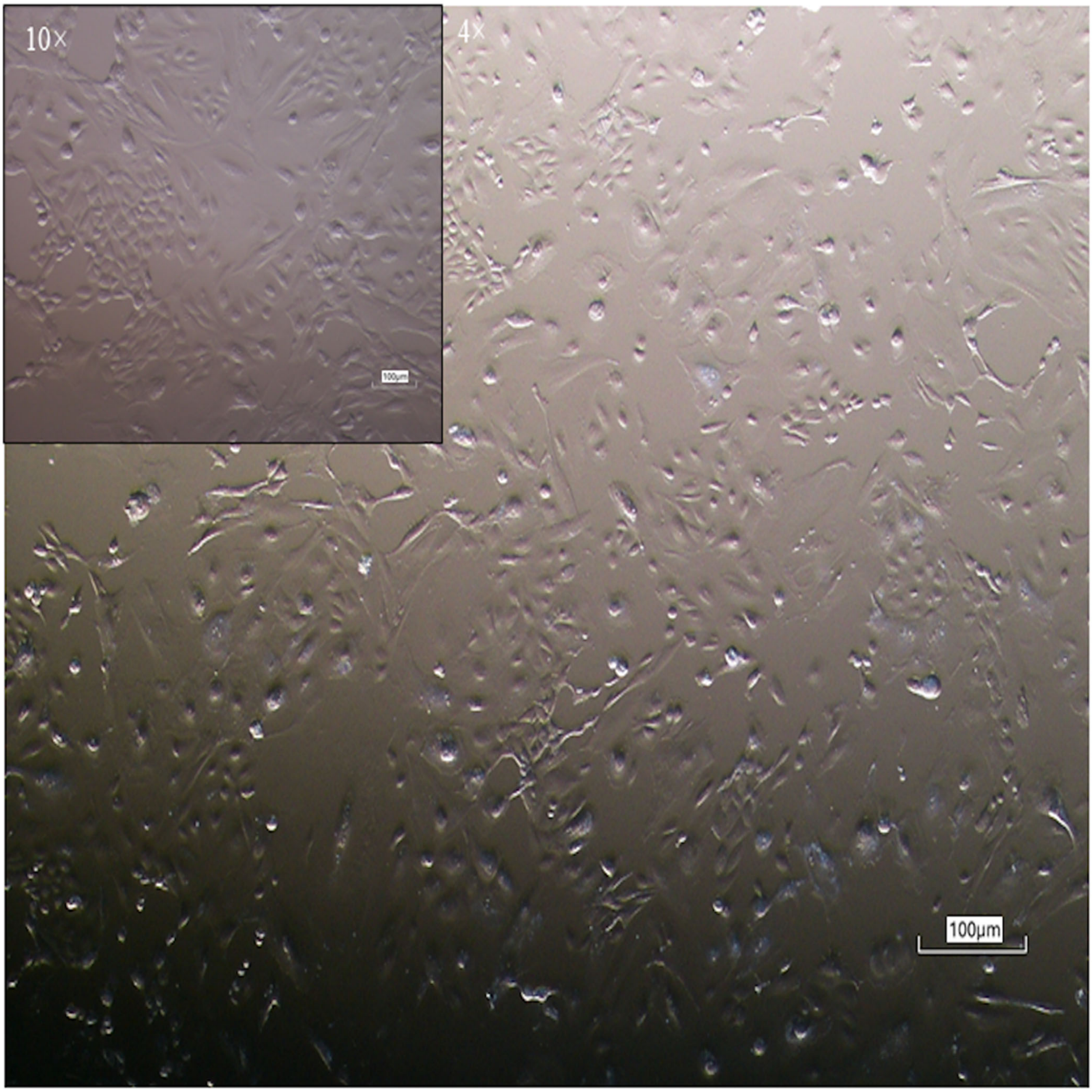

Figure S1. Monolayer of equine colostrum-derived MSCs.

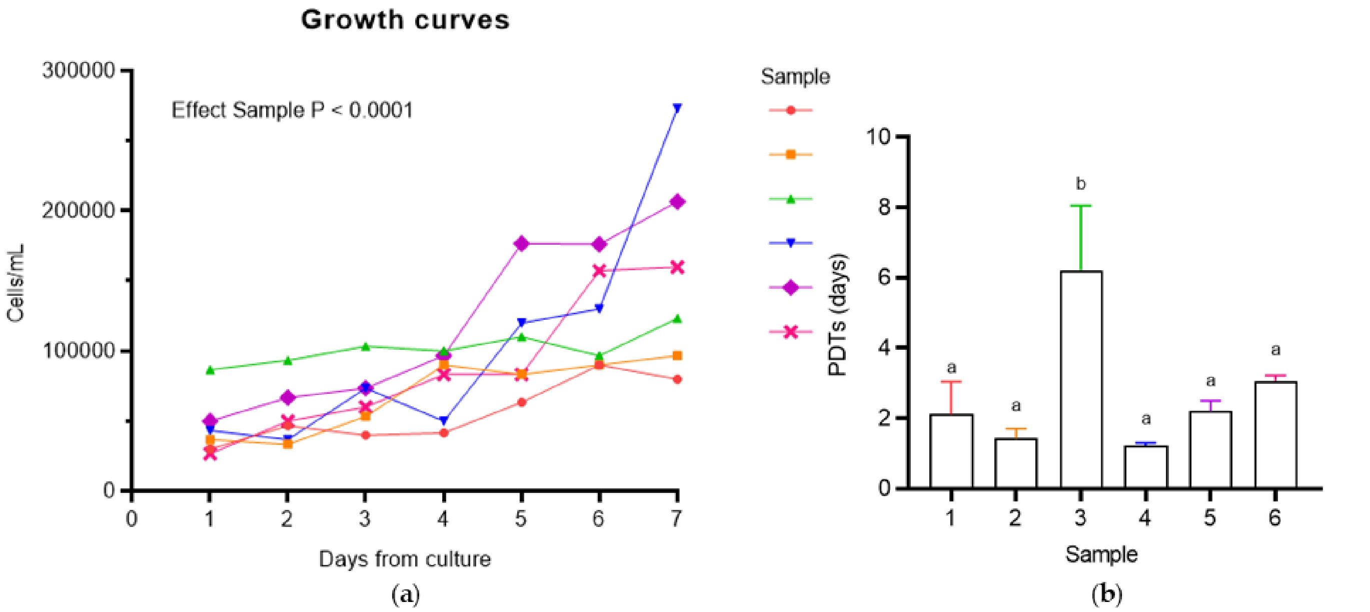

Figure S2. Proliferative capacity of equine colostrum-derived MSCs, evaluated through semi-logarithmic growth curves and population doubling time (PDT) analysis.

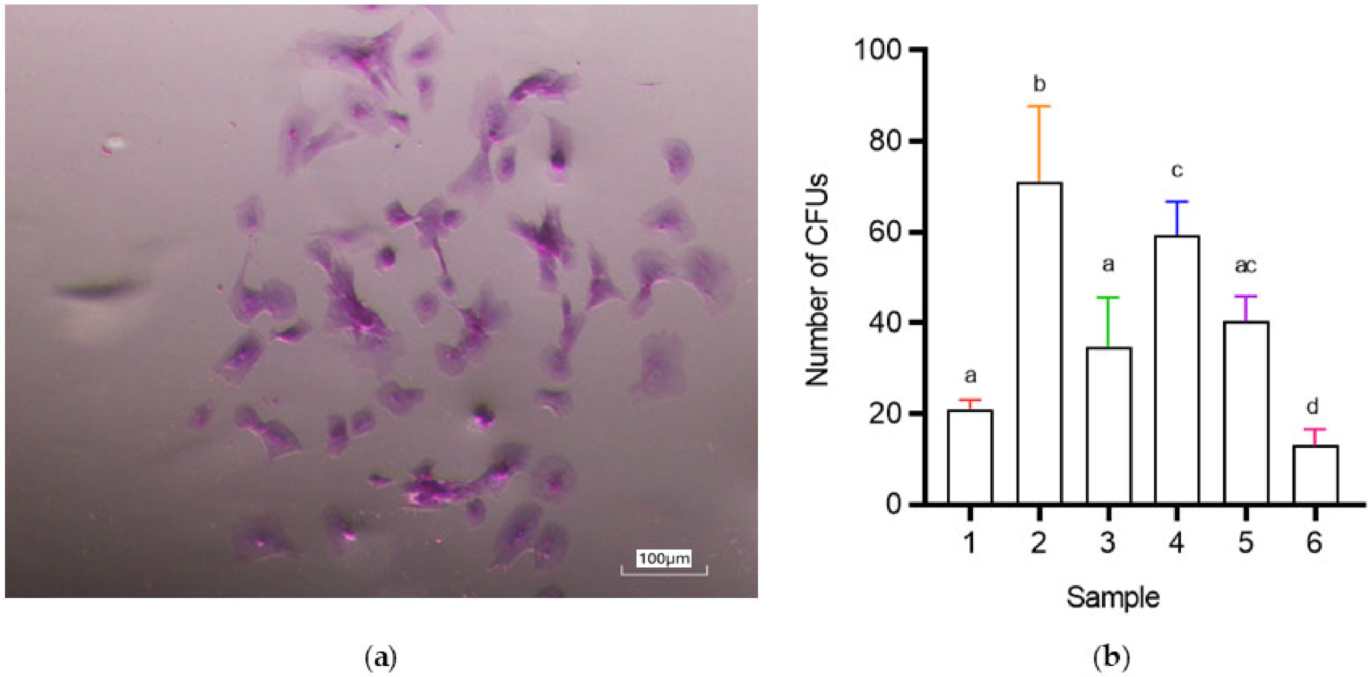

Figure S3. Colony-forming unit (CFU) assay of colostrum-derived MSCs.

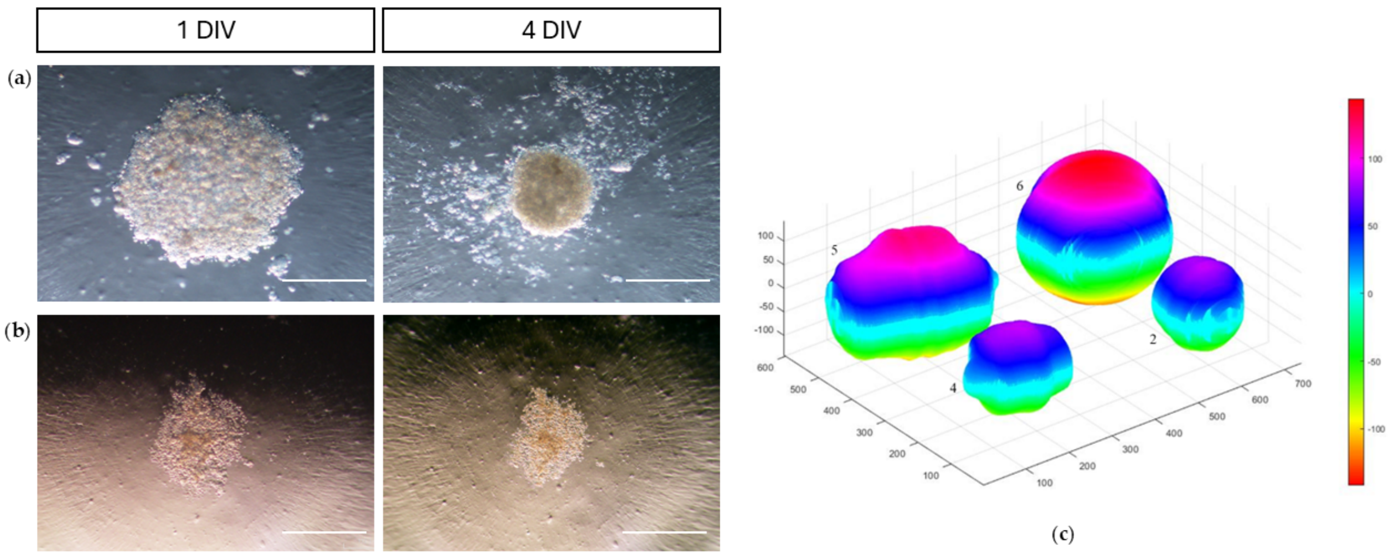

Figure S4. Adhesion assay of colostrum-derived MSCs.

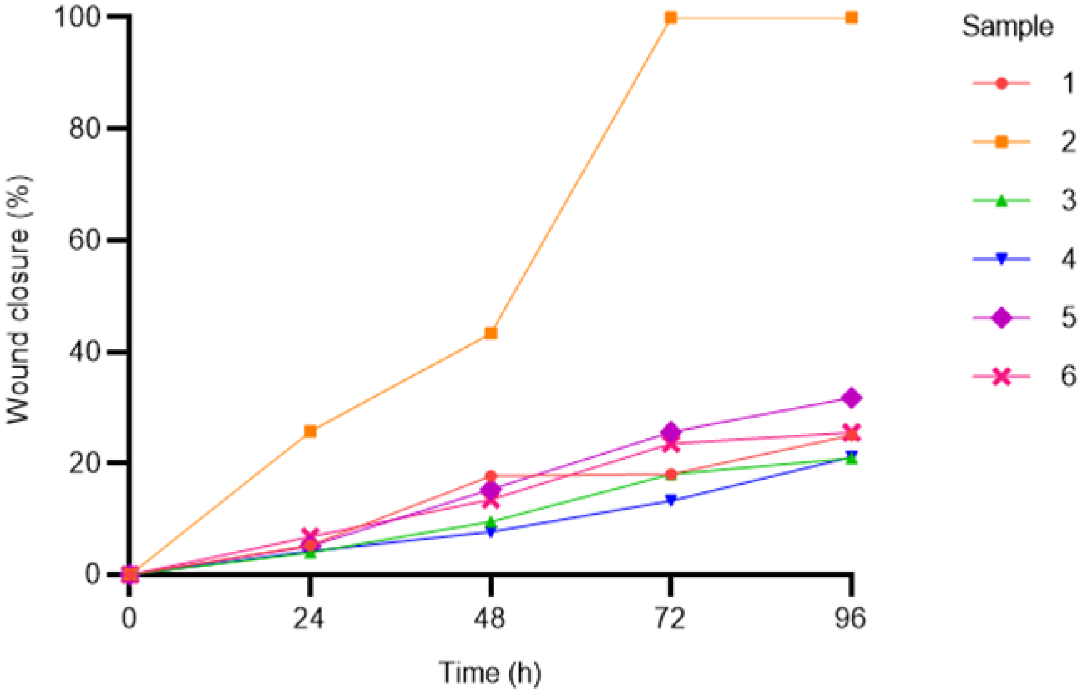

Figure S5. Scratch assay on colostrum-derived MSCs.

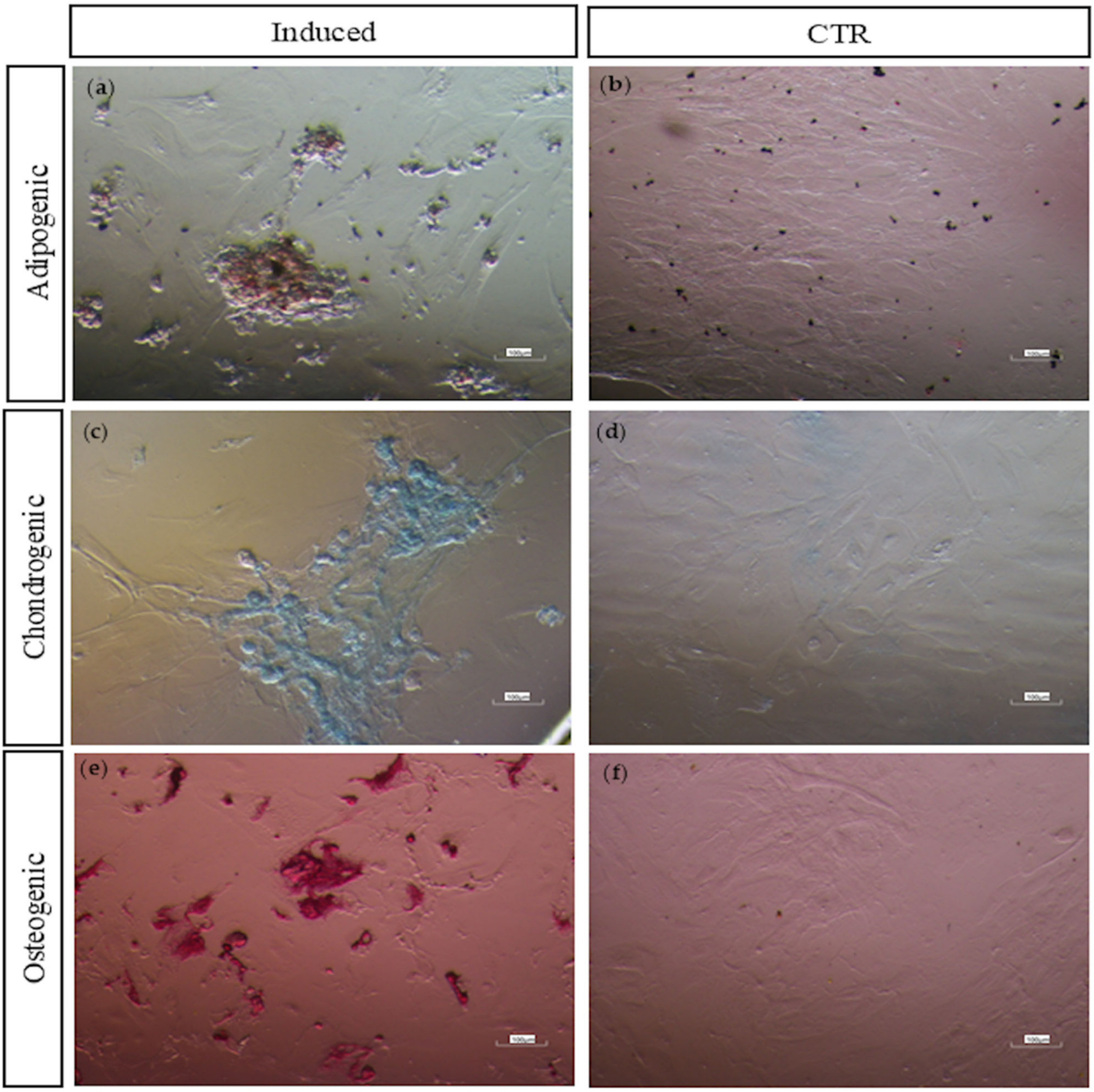

Figure S6. Multilineage differentiation of colostrum-derived MSCs.

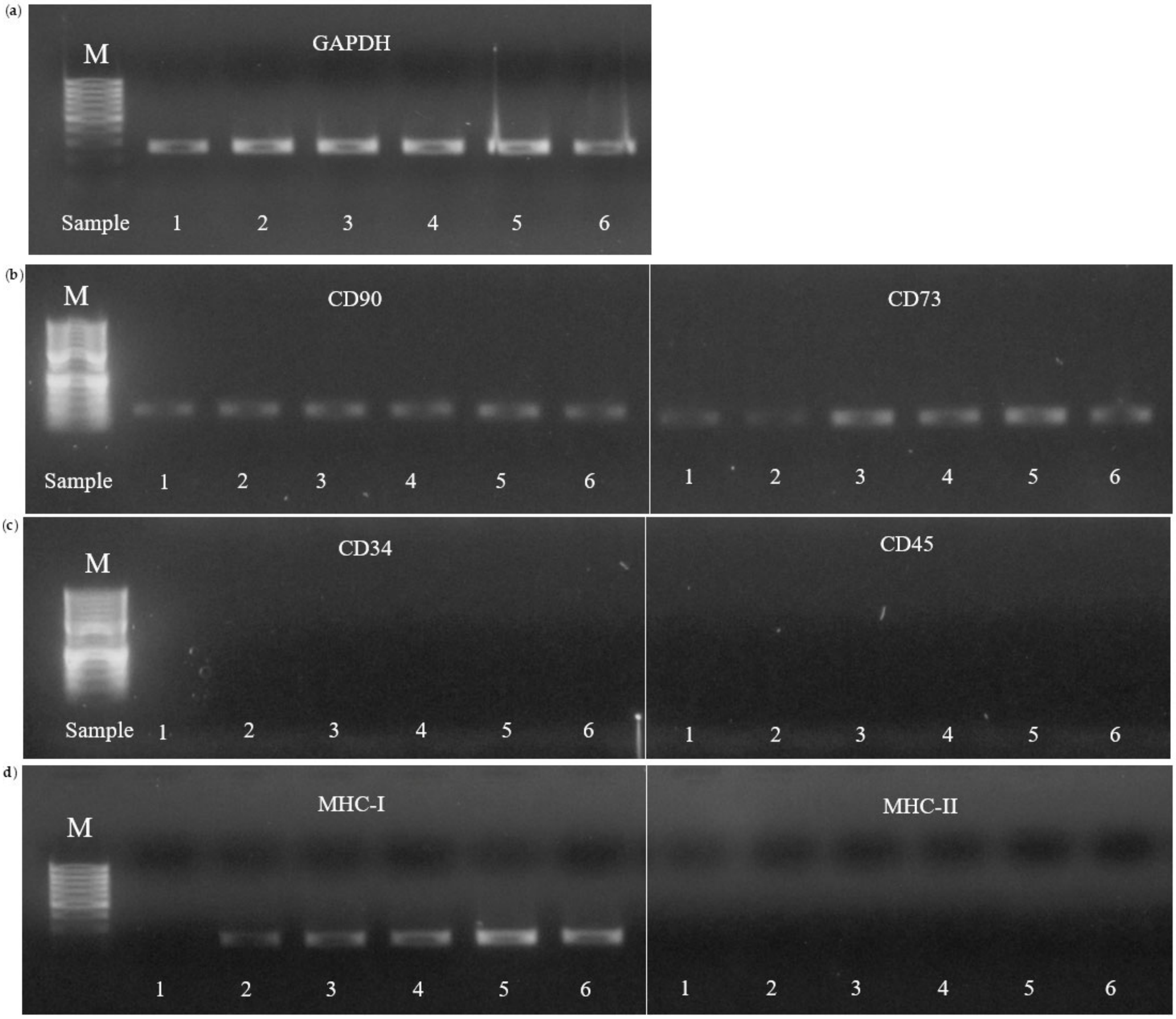

Figure S7. RT-PCR analysis of MSC marker expression in colostrum-derived MSCs.
